# Poly(ε-caprolactone) Titanium Dioxide and Cefuroxime Antimicrobial Scaffolds for Cultivation of Human Limbal Stem Cells

**DOI:** 10.3390/polym12081758

**Published:** 2020-08-06

**Authors:** Mirna Tominac Trcin, Emilija Zdraveva, Tamara Dolenec, Ivana Vrgoč Zimić, Marina Bujić Mihica, Ivanka Batarilo, Iva Dekaris, Valentina Blažević, Igor Slivac, Tamara Holjevac Grgurić, Emi Govorčin Bajsić, Ksenija Markov, Iva Čanak, Sunčica Kuzmić, Anita Tarbuk, Antoneta Tomljenović, Nikolina Mrkonjić, Budimir Mijović

**Affiliations:** 1 The Institute of Immunology, Rockefellerova ul. 2, 10000 Zagreb, Croatia; mirna.tomtrcin@gmail.com; 2 Faculty of Textile Technology, University of Zagreb, Prilaz baruna Filipovića 28a, 10000 Zagreb, Croatia; anita.tarbuk@ttf.unizg.hr (A.T.); antoneta.tomljenovic@ttf.unizg.hr (A.T.); budimir.mijovic@ttf.unizg.hr (B.M.); 3 Department of Transfusion and Regenerative Medicine, Sestre Milosrdnice University Hospital Center, Draškovićeva 19, 10000 Zagreb, Croatia; tamara.dolenec@kbcsm.hr (T.D.); ivana.vrgoc@kbcsm.hr (I.V.Z.); marinabujic@gmail.com (M.B.M.); 4 Department of Microbiology, Croatian Institute for Transfusion Medicine, Petrova 3, 10000 Zagreb, Croatia; ivanka.batarilo@hztm.hr; 5 Medical Faculty of Rijeka, University Eye Hospital Svjetlost, Vjekoslava Heinzela 39, 10000 Zagreb, Croatia; iva.dekaris@svjetlost.hr; 6 School of Medicine, University of Zagreb, Šalata 2, 10000 Zagreb, Croatia; valentina.blazevic@mef.hr; 7 Faculty of Food Technology and Biotechnology, University of Zagreb, Pierottijeva 6, 10000 Zagreb, Croatia; islivac@pbf.hr (I.S.); kmarko@pbf.hr (K.M.); icanak@pbf.hr (I.Č.); 8 Faculty of Metallurgy, University of Zagreb, Aleja narodnih heroja 3, 44000 Sisak, Croatia; tholjev@simet.hr; 9 Faculty of Chemical Engineering and Technology, University of Zagreb, Marulićev trg 19, 10000 Zagreb, Croatia; egovor@fkit.hr (E.G.B.); nikolinamrkonjic1@gmail.com (N.M.); 10 Forensic Science Centre, Ivan Vučetić“, Ilica 335, 10000 Zagreb, Croatia; skuzmic@mup.hr

**Keywords:** scaffolds, tissue engineering, polycaprolactone, electrospinning, titanium dioxide, cefuroxime, limbal stem cell deficiency, antimicrobial activity

## Abstract

Limbal Stem Cell Deficiency (LSCD) is a very serious and painful disease that often results in impaired vision. Cultivation of limbal stem cells for clinical application is usually performed on carriers such as amniotic membrane or surgical fibrin gel. Transplantation of these grafts is associated with the risk of local postoperative infection that can destroy the graft and devoid therapeutic benefit. For this reason, electrospun scaffolds are good alternatives, as proven to mimic the natural cells surroundings, while their fabrication technique is versatile with regard to polymer functionalization and scaffolds architecture. This study considers the development of poly(ε-caprolactone) (PCL) immune-compatible and biodegradable electrospun scaffolds, comprising cefuroxime (CF) or titanium dioxide (TiO_2_) active components, that provide both bactericidal activity against eye infections and support of limbal stem cells growth in vitro. The PCL/CF scaffolds were prepared by blend electrospinning, while functionalization with the TiO_2_ particles was performed by ultrasonic post-processing treatment. The fabricated scaffolds were evaluated in regard to their physical structure, wetting ability, static and dynamic mechanical behaviour, antimicrobial efficiency and drug release, through scanning electron microscopy, water contact angle measurement, tensile testing and dynamic mechanical analysis, antimicrobial tests and UV-Vis spectroscopy, respectively. Human limbal stem cells, isolated from surgical remains of human cadaveric cornea, were cultured on the PCL/CF and PCL/TiO_2_ scaffolds and further identified through immunocytochemistry in terms of cell type thus were stained against p63 marker for limbal stem cells, a nuclear transcription factor and cytokeratin 3 (CK3), a corneal epithelial differentiation marker. The electrospun PCL/CF and PCL/TiO_2_ successfully supported the adhesion, proliferation and differentiation of the cultivated limbal cells and provided the antimicrobial effect against *Pseudomonas aeruginosa, Staphylococcus aureus* and *Candida albicans.*

## 1. Introduction

Tissue damaged in pathological process or after some trauma is usually treated with autografts, allografts or implantable devices. Although autografts are considered the “golden standard” in surgical reconstruction, in terms of low risk of disease transmission and better graft uptake, they still have the risk of a donor-site morbidity [1]. On the other hand, transplantation of allograft depends on donor availability and implies the practice of very stringent tissue banking procedures in order to minimize safety problems and possible immunoreactions. The third solution is implantable medical devices, which have conformational, mechanical, biocompatible as well as lifespan constrains. Tissue engineering is an interdisciplinary field that emerged almost three decades ago with the goal to offer solutions to the issues mentioned above [1]. It concerns the replacement of injured tissues via living cells, temporarily grown onto a scaffolding material. The first examples of these materials included the regeneration of a skin tissue (culturing dermal fibroblasts), cartilage (culturing chondrocytes), as well as an autologous chondrocytes implant material for the replacement of a sternum and so forth. [2,3,4]. Although we are witnessing fast progress in life sciences and technology, tissue engineered products are not yet fully developed to become a part of routine medical treatment. So, the quest of engineering the right scaffold to combine with different cell types and bioactive molecules is still ongoing. Ideally, engineered tissue should acquire architectural complexity of targeted anatomical site. It must be porous enough for the host cells to infiltrate and deposit their own components of extracellular matrix (ECM). Scaffold neovascularization is prerequisite for sufficient exchange of nutrients and metabolites between these cells. There should be adequate cell to cell and cell to ECM interactions [5,6]. Moreover, a scaffold should provide a tissue specific niche for stem cells to grow and exchange activating and inhibitory signals via autocrine, paracrine and endocrine modes [7]. During tissue regeneration process, the scaffold should attract and guide the right types of cells and components of the immune system. The host immunity should promote tissue regeneration without inducing excessive inflammatory response [8]. To achieve some of these properties, researchers are intensively testing various biomaterials made of synthetic or natural materials and their combinations. A proper biomaterial needs to be biocompatible, biodegradable and bioabsorbable. Synthetic materials have several advantages over their natural counterparts. They can be easily shaped in desirable way and biodegraded in controllable fashion. Merging them into composites with varieties of other materials offers even more possibilities to customize them according to individual needs [9,10]. Ability to incorporate specific biomolecules and drugs can further increase their therapeutic efficacy in vivo. Polycaprolactone (PCL) is a synthetic polymer, that has been approved by the Food and Drug Administration (FDA) since the 1970s, for biomedical usage in medical devices, sutures and drug carriers. Since then, it has been gradually replaced with other biomaterials, with more favourable degradation and resorption kinetics. Tissue engineering has brought PCL back in the centre of interest using its favourable characteristics like stability, good rheological and viscoelastic properties [11,12,13]. PCL is stable at room temperature. It is made of hexanoate repeat units and belongs to the family of aliphatic polyesters. This hydrophobic polymer, with semi-crystalline structure, has also a low melting point (59–64 °C) [11,14]. All of these features enable researchers to synthesize PCL economically using often different fabrication techniques. Some of its mechanical, physical and thermal properties can be changed along with its molecular weight [11,12,13]. For example, crystallinity is decreasing with the increase in molecular weight [15]. Moreover, PCL has excellent blending ability with other polymers. Permeability to various compounds makes this polymer a good delivery vehicle for drugs [11,12,13]. One of the latest, most popular technique, that seems promising in the design of an artificial construct that can mimic the natural ECM, is electrospinning. It is a versatile technique that fabricates fibrous structures which are light weight, with high surface-to-volume ratio and adjustable fibres’ diameter/morphology, high porosity and interconnectivity, as well as well-controlled functionality. The principle involves a stretching of an electrically charged viscoelastic polymer solution or melt, that results in the formation of nanofibrous mats collected on a grounded target [16]. In tissue engineering and regenerative medicine, electrospun scaffolds are based on natural polymers like chitosan [17], silk fibroin [18], collagen [19] and so forth but also can be combined with other synthetic biocompatible polymers. These materials are used in the repair of both hard and soft tissues like bones, dental tissues [20], skin, blood vessels, muscles and nerves, cartilage, tendons and ligaments [21]. Functionalized electrospun fibrous scaffolds incorporate specific compounds like drugs, biologically active species as well as other biocompatible chemical agents that can improve the accommodation of specific cell types [22,23,24]. Apart from composition diversity, these structures can provide specific cues for cells surface guidance, based on custom tailored scaffolds topography [25,26], as well as 3D architectures to closely resemble the native tissue microstructures [27,28,29].

In this work we have fabricated and tested scaffold composites made of electrospun PCL and antimicrobial materials like cefuroxime (CF) and inorganic titanium dioxide (TiO_2_) nano and microparticles. Cefuroxime is a semisynthetic antibiotic of the cephalosporin family. It contains a beta-lactam ring attached to a dihyrdothiazole ring. The mechanism of its biocidal action is accomplished through the binding to a specific penicillin-binding proteins, the inhibition of cell walls synthesis and the activation of autolytic enzymes in the bacterial cell wall. Cefuroxime is effective against many beta-lactamase-producing strains. Its broad spectrum of biocidal activity includes anaerobes, gram-negative and gram-positive cocci and bacilli. It is the only cephalosporin from the second generation group that can cross the blood brain barrier. It is often used to treat eye infections caused by bacteria, like *Staphylococcus spp*. and *Streptococcus spp*. For ocular infections, cefuroxime can be applied locally, in the form of drops in aqueous solutions or, artificial tears at concentrations of 1 or 5% [30,31]. Cefuroxime permeates from the conjunctival sac into the aqueous humour and it is less toxic to the corneal endothelium [32]. Titanium dioxide (TiO_2_) is inert and cheap material, with good mechanical and thermal stability. Being nontoxic, it is widely used as a pigment and thickener, in wide range of products for human consumption, from cosmetics and pills, to food additives and tattoo dyes [33,34]. All of these properties along with its antimicrobial activity make this inorganic oxide an interesting material in the medical implants industry and for scaffold engineering [35,36,37,38].

Limbal stem cells (LSCs) are important for homeostasis and repair of the corneal epithelium. The cornea is placed in the frontal segment of the eye. It has a regular and transparent surface, that is very important for light refractive properties. The admitted light is bended on the corneal surface and directed through the pupil in the iris. As the refracted rays of light, traverse the inner structures of the eyeball, they refract again, invert and finally converge in focus on the retina. The image formed there is then converted into electrical signals and transmitted by the optic nerve to the brain [38]. Similar to the skin epithelium, corneal epithelial cells are regularly desquamated and replenished by LSCs from the narrow area, located between the cornea and the sclera, called the limbus. Limbal stem niche within the limbus, possesses a repertoire of molecular signals and structural components, for the LSCs to balance between quiescence, proliferation and differentiation states [39]. Limbal stem cell deficiency is serious condition which can drastically influence the quality of life. It can be caused by congenital conditions, injuries or diseases, which deplete LSCs and damage the limbal niche. Dysfunctional LSCs are no longer able to repair and regenerate the corneal epithelium. Under those circumstances the cornea becomes vascularized, covered with instable conjunctival epithelium, opaque and loses its refractive capability. This leads to a very painful condition with reoccurring corneal defects, ulcers and scarring. Visual function may be seriously damaged. For severe cases of LSC deficiency novel therapeutic treatments are developed. Some of them include ex vivo expansion of LSCs. These cells can be isolated from a small limbal biopsy, harvested from the patient’s healthy eye or from the donor. LSCs can be cultured on several scaffolds like amniotic membrane, fibrin gel or contact lenses [40]. When LSC culture on a scaffold reaches high confluency, the graft is ready for transplantation. Postoperative treatment of the patient’s eye often includes administration of topical antibiotics and corticosteroids [41]. In order to improve clinical outcomes, researchers are trying to develop new scaffolds, that mimic limbal niche properties or incorporate specific drugs. The aim of this study was to assess new scaffolds made of PCL/CF and PCL/TiO_2_ blends for antimicrobial activity and capability to support in vitro growth of LSCs.

## 2. Materials and Methods

### 2.1. Materials and Polymer Solution Preparation

The polymer used as the matrix in this study was polycaprolactone (PCL) with Mn = 80,000 (Sigma Aldrich, St. Louis, MO, USA), while the added component was cefuroxime (CF) (Astro Pharma, Vienna, Austria). The solvents used were glacial acetic acid and acetone (Ru-Ve, Sveta Nedelja, Croatia). Titanium dioxide (TiO_2_), nano (n-TiO_2_ particles 21 nm, Aeroxide P25) and micro (m-TiO_2_ particles 0.1 µm to several µm) powder, were supplied by Sigma-Aldrich, St. Louis, MO, USA. The polymer solution concentration was 18%, while the concentrations of the antibiotic CF were minimum of 5 and maximum of 25 wt %. The blend PCL/CF solution was prepared by dissolving the two components in the blend solvents (volume ratio 8:2). The solution was homogenized by constant stirring, for more than 24 h at room temperature and for up to one hour, with heating at 50 °C when the antibiotic was added.

### 2.2. Electrospun Scaffolds Fabrication and Post-Processing

**Figure 1:**
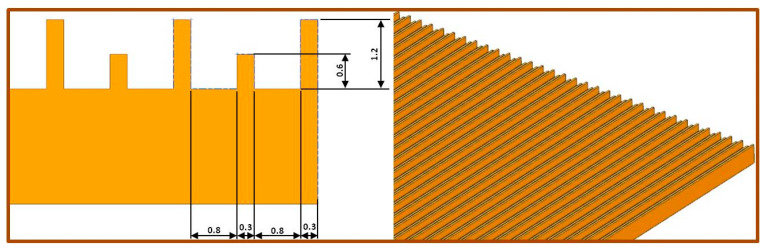
3D printed collector geometry—NX sketch.

The pure PCL and the PCL with the antibiotic were electrospun on a standard electrospinning device, NT-ESS-300, NTSEE Co. Ltd. South Korea, with a modified collector. The collector was prepared on Form 2, 3D printer, FormLabs, with a ribbed geometry (dimensions: ribs’ height 1.2/0.6 mm, ribs’ width 0.3 mm, distance between the ribs 0.8 mm), Figure 1. The processing conditions were—electrical voltage of 14–20 kV, needle tip to collector distance of 18 cm and volume flow rate of 1 mL/h. To prepare the PCL/TiO_2_ scaffolds, 1% nano-TiO_2_ (n-TiO_2_) and micro-TiO_2_ (m-TiO_2_) water solution, was applied on the surface of the electrospun PCL scaffolds postspinning, by an ultrasonic bath, during a sonication time of 60 min.

### 2.3. Characterization of the Electrospun Scaffolds

#### 2.3.1. SEM Analysis and Porosity Calculation

The surface of the PCL, PCL/CF and PCL/TiO_2_ electrospun scaffolds was observed under scanning electron microscopy, Tescan Vega TS 5136 MM and Tescan Mira III, with or without gold coating, at different magnifications. The fibres’ diameters were determined based on the SEM images by measuring 100, randomly selected fibres, using the ImageJ-NIH software. Scaffolds’ porosity was calculated according to Equation (1) [42], where P (%) is the total porosity, m (g) is the sample weight, $A\;\left(cm^{2}\right)$ is the sample area, h (cm) is the sample thickness and $\rho \;\left(\frac{g}{cm^{3}}\right)$ is the polymer density. Samples were evaluated in triplicates. The thickness of the scaffolds was measured by a Digi Micrometer, Mitutoyo.

(1) $$P=\left(1-\frac{m}{A\cdot h\cdot \rho }\right)\cdot 100$$

#### 2.3.2. Water Contact Angle Measurement

To evaluate scaffolds’ wettability a drop of water was placed on the surface of the samples and was imaged with Dino Capture 2.0, Dino-Lite digital microscope, AnMo Electronics. The measurement of the water drop contact angle was conducted by using the Low Bond-Axisymmetric Drop Shape Analysis (LB-ADSA) tool in the ImageJ software [43]. The volume of the water drop was 0.5 mL and the images were taken at 0 and 3–5 s after drop placement. The measurements were carried in triplicates.

#### 2.3.3. Tensile Testing

**Figure 2:**
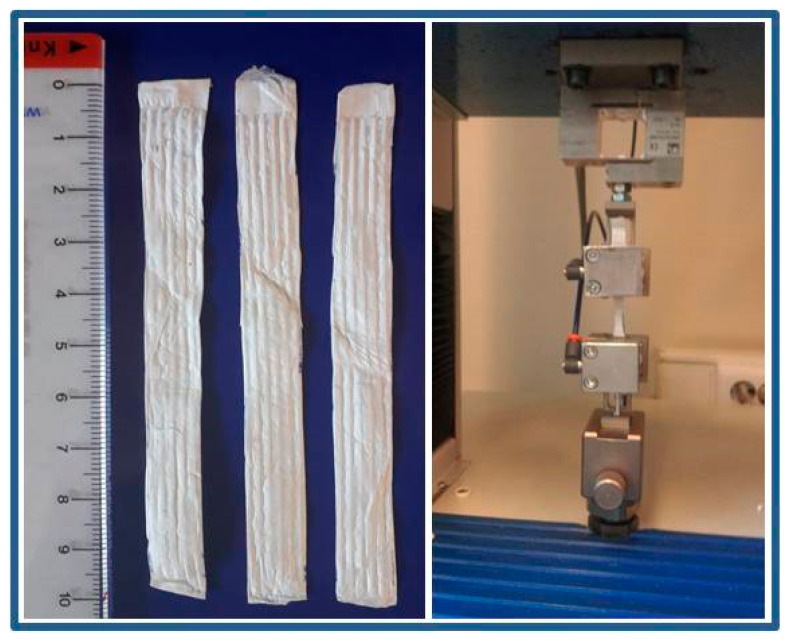
Tensile testing of the electrospun scaffolds.

To evaluate scaffolds’ tensile properties, the samples were tested with Dynamometer Tensolab 3000- tt. Mesdan S.p.A., Italy. The load cell was 100 N, the gauge length was 20 mm and the rate of extension was 20 mm/min. The samples were cut into 10 × 90 mm and were tested in triplicates, Figure 2. Similar testing conditions were reported elsewhere [44].

#### 2.3.4. Dynamic Mechanical Analysis

To determine the viscoelastic properties of the scaffolds, including storage modulus, E’ and loss tangent, tanδ, dynamic mechanical analysis was conducted on DMA 983, TA Instruments. The testing conditions were—frequency of 1 Hz, amplitude of 0.2 mm, heating rate of 3 °C/min and a temperature range between −100 °C and 100 °C. The cooling was conducted under liquid nitrogen.

### 2.4. Antimicrobial Activity of the Electrospun Scaffolds

Two types of antibacterial tests have been conducted against three types of microorganisms—*Pseudomonas aeruginosa, Staphylococcus aureus* and *Candida albicans.* The first one was the disk diffusion test, where a previously grown bacterial culture was spread on a sterile plate, with the disk shaped scaffold in the centre of the plate. After an incubation for 24 h at 37 °C, a zone of inhibition was observed around the tested disks, which size (in mm) is related to the antimicrobial activity level of the scaffolds [45]. Samples were tested in parallel.

In the second test, the bacteria/yeast were cultivated in a Luria-Bertani (LB) broth medium at 37 °C for 24 h. The solution was then diluted to 10^6^ CFU/mL with the LB broth medium. 10 µL of the diluted solution was dropped onto the scaffolds PCL, PCL/5 wt % CF and PCL/m-TiO_2_ in a sterilized Petri dish. The solution containing bacteria/yeast was washed from the samples with 10 mL of LB broth in the Petri dish. 100 µL of each bacteria/yeast suspension solution, was transferred and spread on three nutrient tryptic soy agar (TSA) plates and incubated at 37 °C for 24 h. The average number of surviving colonies on the three incubated agar plates was further calculated [46]. The number of tested samples, per each scaffold type, was 15 for each of the bacteria (*Pseudomonas aeruginosa* and *Staphylococcus aureus*), while 10 samples per each scaffold type for the *Candida albicans*.

#### UV-Vis Spectroscopy—Active Components Release Profiles

The release profile of the antibiotic in the PCL/CF scaffolds was observed under UV/Vis transmission spectrophotometer, Cary 50, Varian. The samples were cut into 20 × 20 mm and incubated in 10 mL of phosphate-buffered saline (PBS) solution at 37 °C (with shaking at 80 rpm) for a period of 14 days. Every 24 h, 2–3 mL of the solutions were taken and recorded with the spectrophotometer. The solutions were returned back to the bulk, as to maintain the same volume during the testing period. The concentrations of the released antibiotic were calculated based on a standard curve fitted among the known concentrations, of the dissolved CF into the PBS, ranging from 0.007 to 0.05 mg/mL.

### 2.5. Human LSCs Culture and Characterization on the Electrospun Scaffolds

#### 2.5.1. Preparation of 3T3 Feeder Layer

Cultivation of 3T3 mouse cells and preparation of a feeder layer was carried as follows, the 3T3 cells (ATCC-CCL-92, Swiss albino) were cultured in growth medium, containing Dulbecco’s Modified Eagle Medium (DMEM) (Gibco, Invitrogen, by Thermo Fisher Scientific, Waltham, MA, USA), 10% of heat inactivated Foetal Bovine Serum (FBS) (Gibco, Invitrogen, by Thermo Fisher Scientific, Waltham, MA, USA), antibiotic-antimycotic (ABAM) and 1% L-glutamine. Trypsinized 3T3s were neutralized with the medium containing FBS and treated with γ-rays to inhibit any further proliferation. The dosage used maintained them metabolically active for several days. 

#### 2.5.2. Isolation and Cultivation of the LSCs

Human LSCs were isolated from the surgical remains of human cadaveric cornea, with prior permission of the Ethics Committee of the University Eye Hospital Svjetlost (Zagreb, Croatia). The scaffolds PCL, PCL/CF and PCL/TiO_2_ were cut in circles, with 15 mm diameter and disinfected for 15 min on each side under the UV-light in a microbiological safety cabinet. The scaffolds were put in 24 well plates and seeded with irradiated 3T3s in the concentration of 1.5 × 10^4^ cells/cm^2^ per well. LSCs of the second passage were subsequently seeded on the feeder cells in 1:1 ratio. The keratinocyte growth medium containing 10% FBS, 2:1 DMEM—Ham’s F-12 (Invitrogen, by Thermo Fisher Scientific, Waltham, MA, USA), 2% L-glutamin, 1% ABAM, 5 µg/mL insulin (Sigma-Aldrich, St. Louis, MO, USA), 0.18 mM adenine (Sigma-Aldrich, St. Louis, MO, USA), 0.4 µg/mL, hydrocortisone (Sigma-Aldrich, St. Louis, MO, USA), 0.1 nM cholera toxin (Accurate Chemicals, Westbury, NY, USA), 2 nM triiodothyro-nine (Sigma-Aldrich, St. Louis, MO, USA) and 10 ng/mL epidermal growth factor (EGF) (Sigma-Aldrich, St. Louis, MO, USA) was changed every third day until LSC culture reached 70%–100% confluence.

#### 2.5.3. Immunocytochemistry and Confocal Imaging of the LSCs Cultured on the Electrospun Scaffolds

Indirect immunocytochemistry (ICC) was performed on the scaffolds cultivated with LSCs. The cells were fixed in 4% paraformaldehyde (Sigma-Aldrich, St. Louis, MO, USA) for 10 min and permeabilized with 0.5% triton X-100 (Sigma-Aldrich, St. Louis, MO, USA). Incubation with primary antibodies was carried out overnight at +4 °C. The primary antibodies used were mouse monoclonal antibody [4A4] IgG2a to human p63 protein (Abcam, Cambridge, UK), diluted in the ratio 1:100 and mouse monoclonal antibody [AE5] IgG1 to human cytokeratin 3/CK-3 (Abcam, Cambridge, UK), diluted in the ratio 1:50. Secondary antibodies used were goat anti-mouse IgG Alexa Fluor 488 (Invitrogen, by Thermo Fisher Scientific, Waltham, MA, USA), diluted in phalloidin-tetramethylrhodamine B isothiocyanate (TRITC) (Sigma-Aldrich, St. Louis, MO, USA) and goat anti-mouse IgG Alexa Fluor 568 (Invitrogen, by Thermo Fisher Scientific, Waltham, MA, USA), both in concentration of 5 µg/mL. The secondary antibodies labelled with fluorochromes, were incubated under dark conditions for 30 min at 37 °C. The cell nuclei were marked with 4′,6-diamidino-2-phenylindole (DAPI) (Sigma-Aldrich, St. Louis, MO, USA). Before the microscopy analysis, samples were mounted with Prolong Antifade Kit (Invitrogen, by Thermo Fisher Scientific, Waltham, MA, USA) and stored at −20 °C. Confocal microscopy was carried out on Leica, TCS SP2 AOBS (Leica Microsystems CMS GmbH, Mannheim, Germany) at the Ruder Bošković Institute (Zagreb, Croatia). 

#### 2.5.4. Metabolic Activity of the LSCs on the Electrospun Scaffolds

Nine days after seeding, an 3-(4,5-dimethylthiazol-2-yl)-2,5-diphenyltetrazolium bromide (MTT) assay was performed to test the presence of living cells on the electrospun scaffolds by 3- (4,5-dimethylthiazol-2-yl)-2,5-diphenyltetrazole bromide staining. This test quantitatively determines the biocompatibility of the tested scaffolds through cell viability expressions. The MTT solution is added to the wells with the seeded scaffolds and the staining is first detected visually (live cells convert the yellow MTT into dark blue formazan) four hours after incubation at 37 °C, followed by dimethyl sulfoxide (DMSO) treatment and spectrophotometric measurement of the absorbance at 570 nm. The results were expressed as a relative ratio of the number of living cells on the scaffolds, to the negative control (wells with no scaffolds but with identical cell inoculum). 

#### 2.5.5. Statistical Analysis

The results in the study are expressed as average ± standard deviation (SD) values. Analysis of variance (ANOVA one-way) and Tukey post-tests were performed in Origin8, to compare means between groups and were considered significantly different at the level of 0.05.

## 3. Results

### 3.1. Electrospun Scaffolds Morphology and Total Porosity

**Figure 3:**
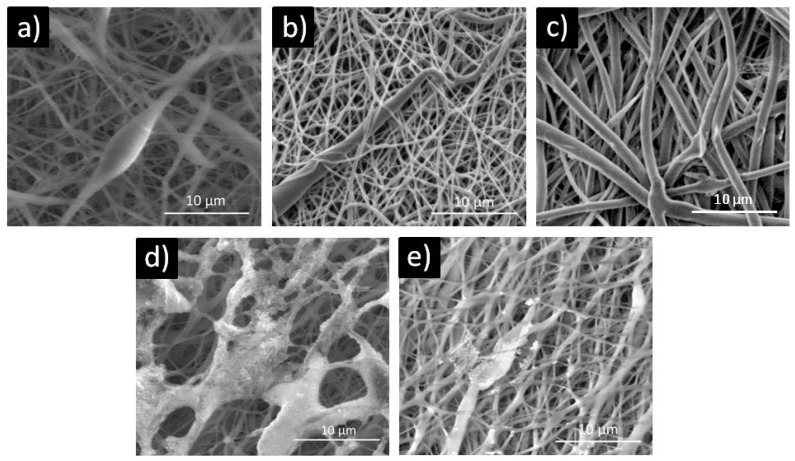
Scanning electron microscopy (SEM) photomicrographs of the electrospun scaffolds: (**a**) poly(ε-caprolactone) (PCL) with randomly beaded fibers, (**b**) PCL/5 wt % CF and (**c**) PCL/25 wt % CF with uniform fibers, (**d**) PCL/n-TiO_2_ and (**e**) PCL/m-TiO_2_ with surface particle agglomerations.

Figure 3 gives the scanning electron microscopy (SEM) photomicrographs of the electrospun PCL, PCL/5 wt % CF, PCL/25 wt % CF, PCL/n-TiO_2_ and PCL/m-TiO_2_ scaffolds.

The pure PCL fibres, Figure 3a, were noticed to have random beads (deformations) along the lengths, while the addition of the antibiotic resulted in their removal, Figure 3b,c. The nano and micro TiO_2_ coatings on the surface of the PCL were generally noticed in the form of agglomerations especially in case of the PCL/n-TiO_2_, Figure 3d,e. These scaffolds’ fibres morphology was not changed drastically but only random fibres agglutinations were present due to the sonication treatment.

**Figure 4:**
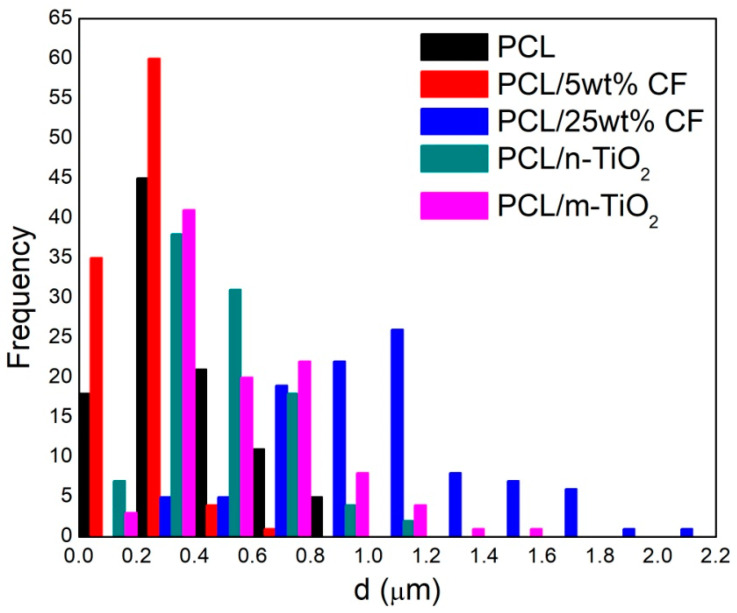
Fiber diameter distribution of the electrospun scaffolds.

Figure 4 gives the distributions (histograms) of each of the electrospun scaffolds fibres’ diameters. The diameters ranges were almost overlapping for the electrospun PCL and PCL/5 wt % CF (~90–950 nm), as well as the PCL/n-TiO_2_ and PCL/m-TiO_2_ scaffolds (~100 nm to 1.6 μm). The mean fibre diameters of the electrospun PCL, PCL/5 wt % CF, PCL/25 wt % CF, PCL/n-TiO_2_ and PCL/m-TiO_2_ scaffolds were as follows, 0.374 ± 0.202 μm, 0.241 ± 0.197 μm, 1.00 ± 0.362 μm, 0.450 ± 0.206 μm and 0.516 ± 0.258 μm, respectively. ANOVA one-way and Tukey tests revealed mean values of the fibres’ diameters significantly different at the level of 0.05 between the PCL and the PCL/5 wt % CF, PCL/25 wt % CF and PCL/m-TiO_2_ scaffolds, while there was no significant difference between the PCL and the PCL/n-TiO_2_ electrospun scaffolds, at the level of 0.05.

**Figure 5:**
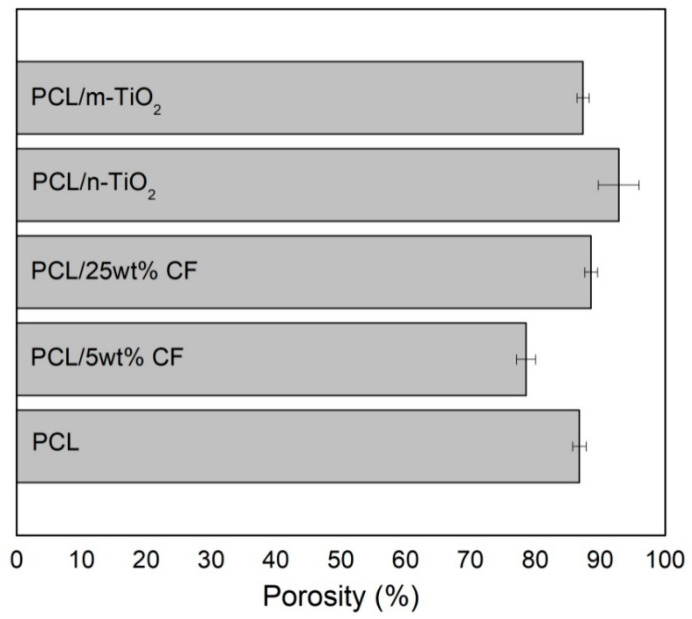
Electrospun scaffolds total porosity.

As for the scaffolds total porosity, Figure 5, the calculated porosities were in the ranges between 78.58 ± 1.45 up to 92.87 ± 3.13%. Statistical analysis showed significantly different values at the level of 0.05 between the PCL and the PCL/5 wt % CF and PCL/n-TiO_2_ scaffolds, while no significant difference was in comparison with the PCL/25 wt % CF and the PCL/m-TiO_2_ scaffolds.

### 3.2. Electrospun Scaffolds Surface Wettability

**Figure 6:**
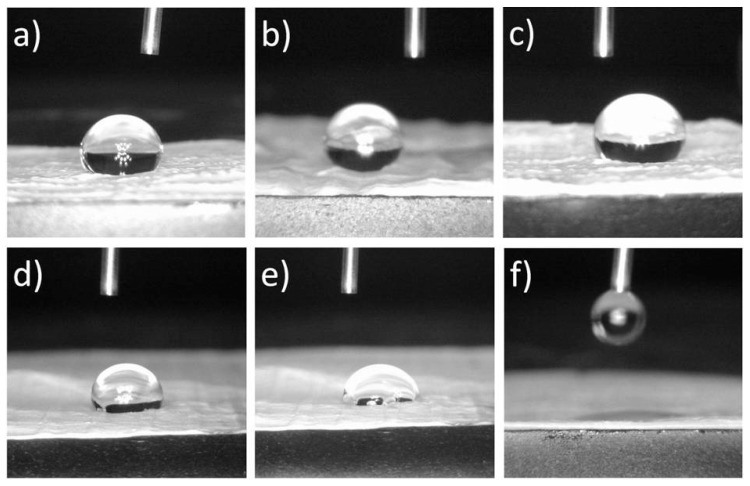
Water contact angle measurement on the surface of the electrospun scaffolds: (**a**) PCL, (**b**) PCL/n-TiO_2_, (**c**) PCL/m-TiO_2_, (**d**) PCL/5 wt % CF (1 s), (**e**) PCL/5 wt % CF (5 s), (**f**) PCL/25 wt % CF.

The electrospun scaffolds were evaluated for their surface wettability by measuring the water contact angle on the surface of the samples. Figure 6 shows the water drops on the surface of the scaffolds and Table 1 shows the mean values of the measured water contact angles. In the first second of measurements, there was no significant difference at the level of 0.05, between the values of the water contact angles of the PCL and all other scaffolds, except for the PCL/25 wt % CF scaffold. As a hydrophobic polymer the pure PCL shows very high water contact angle, Figure 6a, almost 118°, with no change of the same with time. Both nano and micro TiO_2_ application had no effect on the surface wettability, Figure 6b,c, but slightly increased the water contact angle compared to the pure PCL. On the other hand, after several seconds, significant difference at the level of 0.05 is noticed for the wettability between the PCL and the PCL/CF scaffolds. Thus, a hydrophilic effect was evident in case of the antibiotic addition, where the 5 wt % of the CF, Figure 6e, resulted in a water contact angle lower than 90° after 5 s, while the 25 wt % of the CF, Figure 6f, showed immediate water drop absorption which suggests high wetting effect. Similarly, in a study of PCL electrospun scaffolds modified with ECM for corneal stroma regeneration, the pure PCL scaffolds were reported to have a water contact angle of 121.2° ± 2.0°, while the addition of the ECM had a reduction effect of this value [47]. Another study discussed the increase in PCL surface wettability with the incorporation of metformin hydrochloride or metoprolol tartrate drugs by blend electrospinning. The reduced angles were from 136.47° ± 0.84° to 127.83° ± 1.04° and 114.70° ± 2.56°, respectively or to zero in case of emulsion electrospinning [48]. 

### 3.3. Tensile Properties of the Electrospun Scaffolds

**Figure 7:**
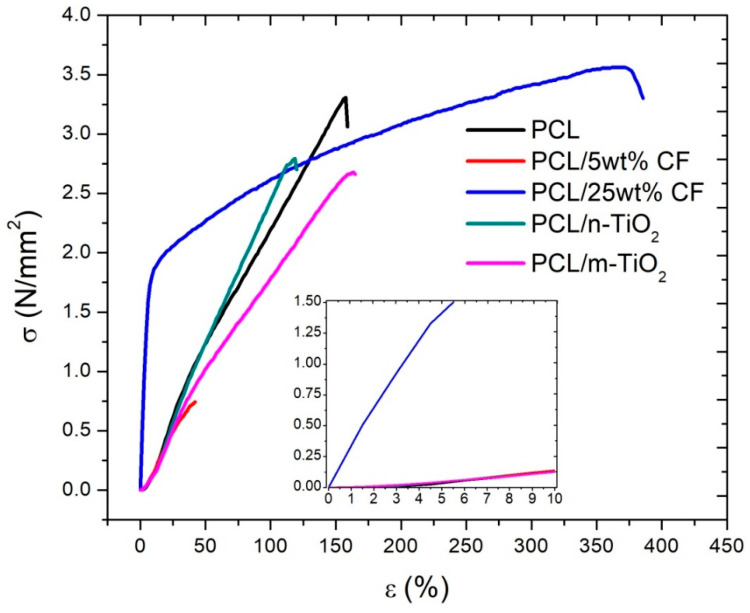
Tensile stress-strain curves of the electrospun scaffolds.

The mechanical properties are also affected by the added components in the polymer (matrix) of the scaffolds. Figure 7 gives the tensile stress-strain curves, while Table 2 gives the maximum force, elongation at break and tensile strength of the electrospun scaffolds. The tensile behavior of the scaffolds, with both the added nano and micro TiO_2,_ was not significantly different compared to the pure PCL scaffolds, although both components reduced the ultimate strain and tensile strength of the scaffolds. Statistical analysis confirmed that there is a significant difference at the level of 0.05 concerning tensile strength only between the PCL and the PCL/5 wt % CF scaffolds, while in terms of elongation at break this is in case of the PCL and both CF based electrospun scaffolds. Thus, the addition of the antibiotic showed significantly different stress-strain curves, as in case of the PCL/25 wt % CF, a very high value of the elastic limit was noticed. The PCL/25 wt % CF scaffold ultimate strain was more than twofold greater than that of the pure PCL. The results obtained in this study are comparable to other similar studies concerning tissue engineering application. For example, authors reported on the development of PCL/chitosan-gelatin complex scaffolds with a tensile strength in the range of 2.24 ± 0.35 to 17.6 ± 0.51 MPa depending on the chitosan-gelatin content, thus resulting in a variable effect when compared to the tensile strength of the pure PCL (5.52 ± 0.07 MPa) [49]. Another study, considered a combination of PCL with gelatin only, to be used for the propagation of limbal epithelial stem cells, in the repair of an animal model cornea. The mechanical performance of the scaffolds revealed 1.7 MPa of the pure PCL, while the blend PCL-gelatin scaffold showed an increase of the tensile strength of more than 2.5 MPa [50].

**Figure 8:**
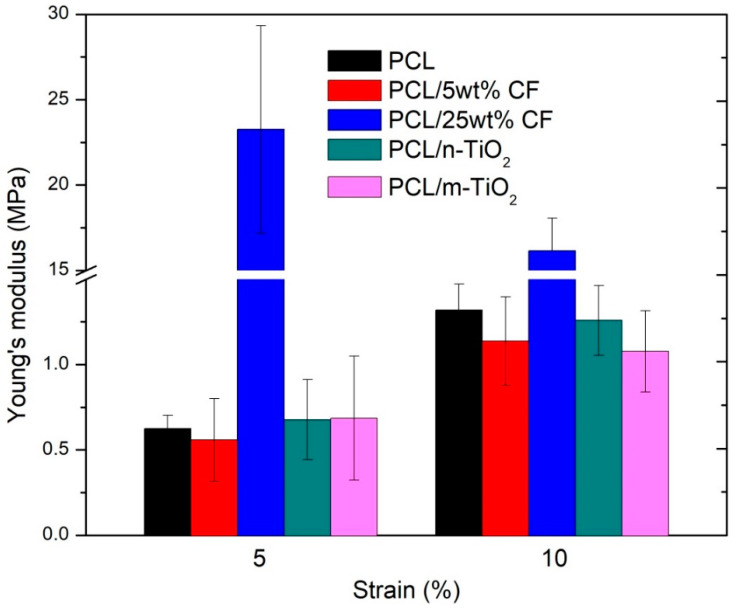
Young’s moduli of the electrospun scaffolds.

Figure 8 gives the calculated Young’s moduli of the electrospun scaffolds at the strains of 5 and 10%, which were taken as the limit values of the elastic region for the PCL/25 wt % CF and all other scaffolds, respectively. The highest Young’s modulus (23.3 ± 6.1 MPa) was in case of the electrospun PCL/25 wt % CF at the strain of 5%, while all other scaffolds at the same strain showed very low values of around 0.7 MPa. At the strain of 10% all scaffolds showed higher Young’s modulus thus still remaining relatively elastic, while the electrospun PCL/25 wt % CF after the 5% strain becomes plastic, which is evident from the shallow slope of the σ-ɛ curve, Figure 7. Statistical analysis of the results confirmed that a significant difference at the level of 0.05 of the Young’s moduli, was observed for the pure PCL and the PCL/25 wt % CF scaffold at both 5 and 10% levels of the strain, while the same is evident between the PCL/25 wt % CF and all other scaffolds. Study that considers the regeneration of the corneal stromal tissue, reported on the gelatin scaffold Young’s modulus to be 136 ± 61 MPa, while with the addition of PCL it reduced down to 15 ± 5 MPa, when the gelatin content was zero [51]. For comparison, scaffolds based on gelatin/PCL and collagen/poly(l-lactic acid-co-ɛ-caprolactone), for vascular tissue engineering, were reported to have Young’s moduli of 1.49 ± 0.06 and 1.77 ± 0.09 MPa, respectively [52]. Variation of the mechanical parameters among studies is certainly related to scaffolds’ composition in terms of polymers, fillers as well as solvents used but also in terms of processing parameters that influence fibers morphology, thickness, porosity, fibers collection (orientation) and so forth.

### 3.4. Viscoelastic Properties of the Electrospun Scaffolds

**Figure 9:**
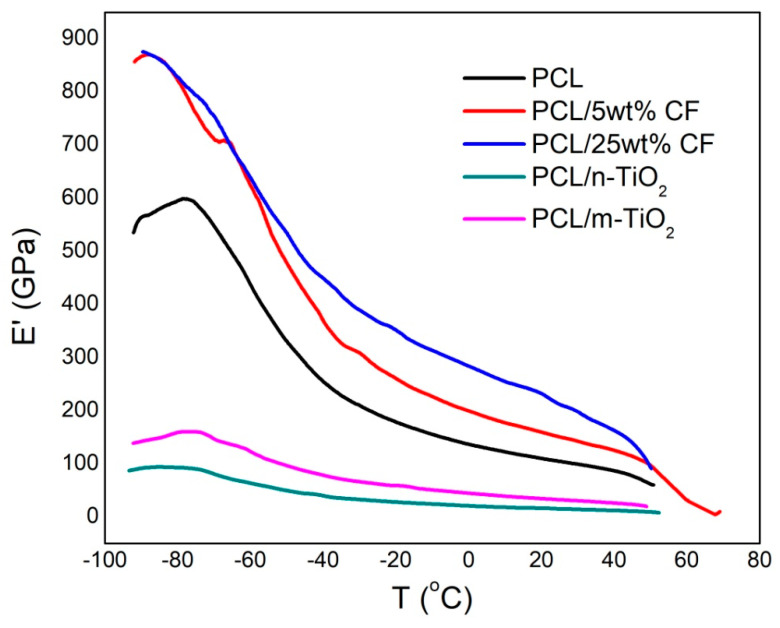
Storage modulus as a function of temperature of the electrospun scaffolds.

**Figure 10:**
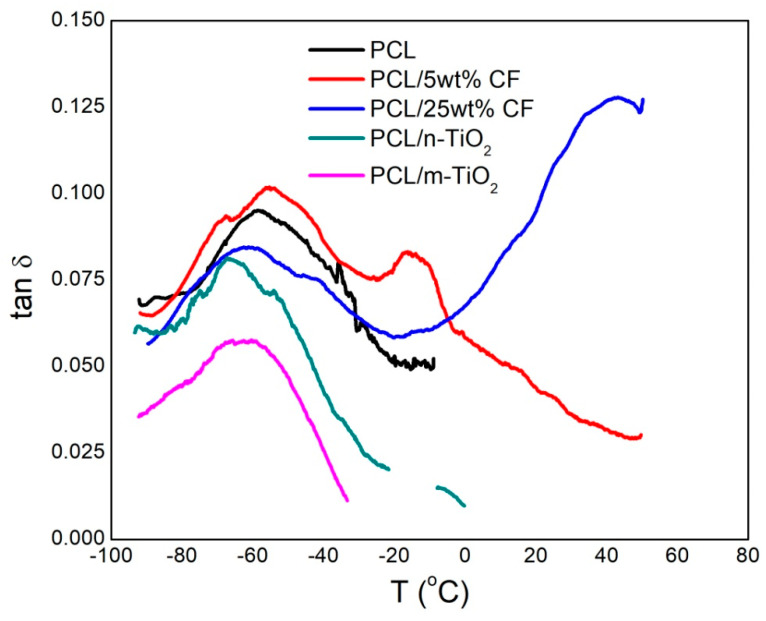
Damping as a function of temperature of the electrospun scaffolds.

The viscoelastic properties of the electrospun PCL, antibiotic and TiO_2_ based scaffolds were determined by dynamic mechanical analysis. The effect of the additives on the pure PCL performance was examined by measuring the storage modulus (E’) and damping (tan δ) as a function of temperature in the range of −100 to 80 °C, Figure 9 and Figure 10.

The added nano and micro TiO_2_ particles were not acting as nucleating agents thus showed reduced stiffness of the scaffolds when compared to the pure PCL, Figure 9. These scaffolds showed slight changes in the relaxation maximum, Figure 10, which is related to the glass transition temperature (T_g_) of the PCL’s amorphous phase. Greater shift of the T_g_ to lower temperature was observed for the PCL/n-TiO_2_ scaffold thus affecting the PCL chain movement. Slightly higher T_g_ in case of the PCL/5 wt % CF scaffold results in reduced PCL’s amorphous phase mobility.

The shift in the T_g_ and its effect on the amorphous PCL phase noticed in case of the added antibiotic CF is due to its crystalline structure, which finally resulted in the increase of the scaffold stiffness compared to all other scaffolds.

### 3.5. Antimicrobial Efficiency of CF and TiO_2_ Components in the Electrospun Scaffolds

The antimicrobial tests conducted against three types of microorganisms including, *Pseudomonas aeruginosa, Staphylococcus aureus* and *Candida albicans*, confirmed the antimicrobial efficiency for both PCL/TiO_2_ and PCL/CF electrospun scaffolds.

**Figure 11:**
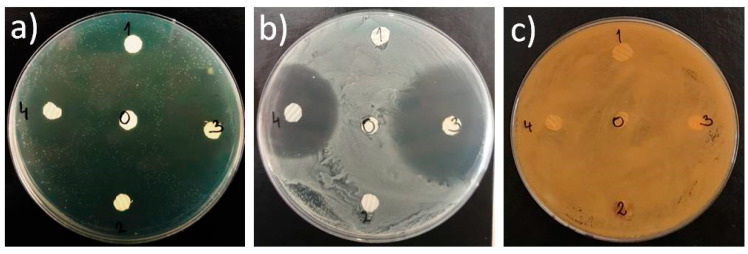
Antimicrobial activity of the electrospun scaffolds against: (**a**) *Pseudomonas aeruginosa* 3024, (**b**) *Staphylococcus aureus* 3048 and (**c**) *Candida albicans* 11.

In the disk diffusion test, Figure 11, both PCL/CF concentrations and the PCL/n-TiO_2_ scaffolds developed inhibition zones, Table 3, against *Staphylococcus aureus* and *Pseudomonas aeruginosa* with the highest activity level in case of the 25 wt % of the CF antibiotic (zone greater than 40 mm). The PCL/m-TiO_2_ scaffold has not proven its antimicrobial activity with the disk diffusion test.

**Figure 12:**
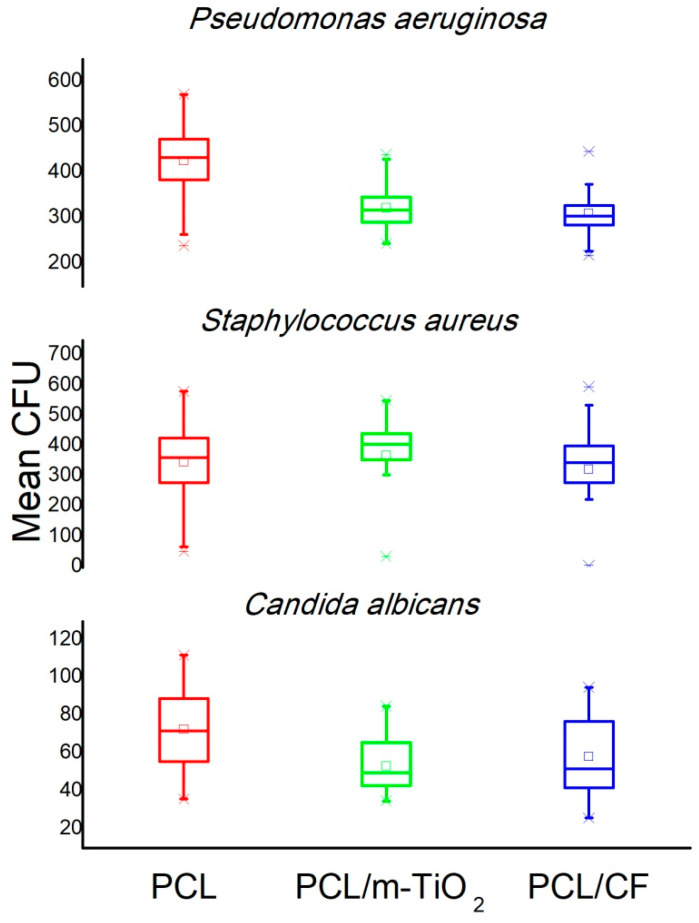
Antimicrobial efficacy of the electrospun PCL, PCL/m-TiO_2_ and PCL/5 wt % CF scaffolds against *Pseudomonas aeruginosa*, *Staphylococcus aureus* and *Candida albicans*.

In the second test, where the average number of surviving colonies was calculated, both the CF antibiotic and the micro TiO_2_ particles confirmed their antimicrobial activity as the colony forming units (CFU) were lower when compared to the pure PCL scaffold, with an exception for the PCL/m-TiO_2_ in case of the *Staphylococcus aureus*. ANOVA one way and Tukey tests have revealed mean CFU values significantly different at the level of 0.05 in case of the PCL, PCL/m-TiO_2_ and PCL/CF electrospun scaffolds when tested against *Pseudomonas aeruginosa* and *Candida albicans*, Figure 12.

### 3.6. Evaluation of the Antimicrobial Components Release Profiles

The release profiles of the antimicrobial components from the electrospun PCL/CF and PCL/TiO_2_ scaffolds were determined by UV measurement of the incubated PBS/samples solutions’ absorbances, every 24 h. It is important to highlight that no absorbance peaks were detected in case of the TiO_2_ components, over the total time period. This suggests that the released amounts of the TiO_2_ dispersions were very low to be detected.

**Figure 13:**
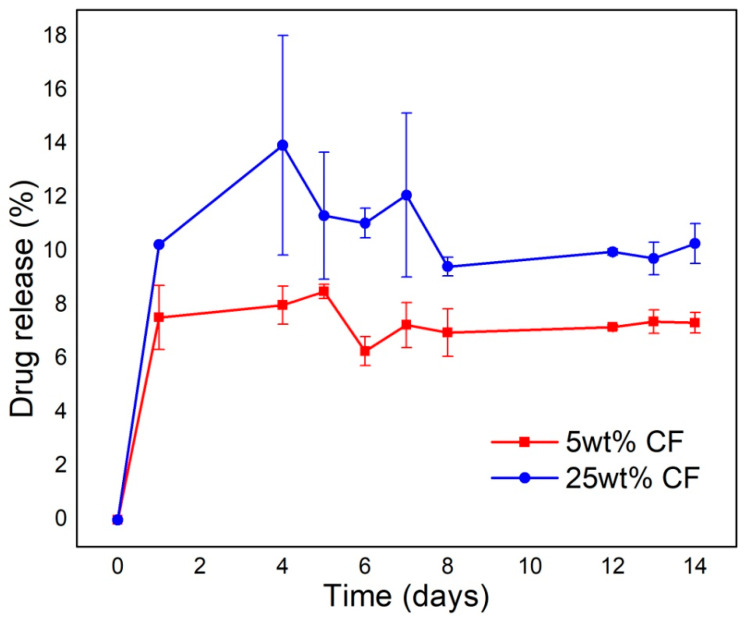
Release of the cefuroxime in percentages from the electrospun PCL/CF scaffolds.

Figure 13 shows the release profile of the cefuroxime, concerning both concentrations of 5 and 25 wt % CF, during a period of 14 days, which is chosen based on an average posttransplant antibiotic treatment duration. As expected, the scaffold with the initial higher CF content showed higher overall drug release%. Generally, both CF profiles were quite similar, thus with an initial burst release stage after the first 24 h to day 4, followed by an almost sustained release to day 14. The maximum release content of the drug was 14% and almost 9%, for the 25 wt % and the 5 wt % CF, respectively.

### 3.7. SEM Analysis and Metabolic Activity of the LSCs on the Electrospun Scaffolds

**Figure 14:**
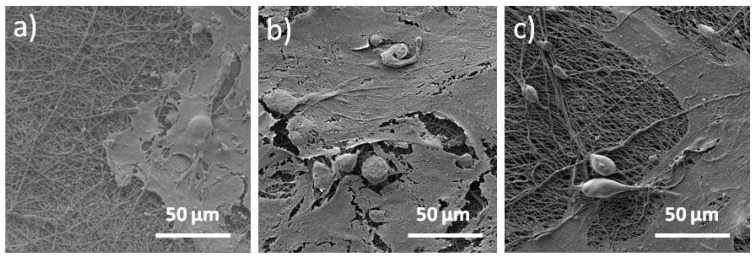
SEM photomicrographs of the adhered Limbal stem cells (LSCs) on the electrospun scaffolds: (**a**) PCL, (**b**) PCL/5 wt % CF and (**c**) PCL/m-TiO_2_.

Representative SEM photomicrographs that confirm the adhesion of the limbal stem cells on the electrospun scaffolds surfaces are given in Figure 14.

**Figure 15:**
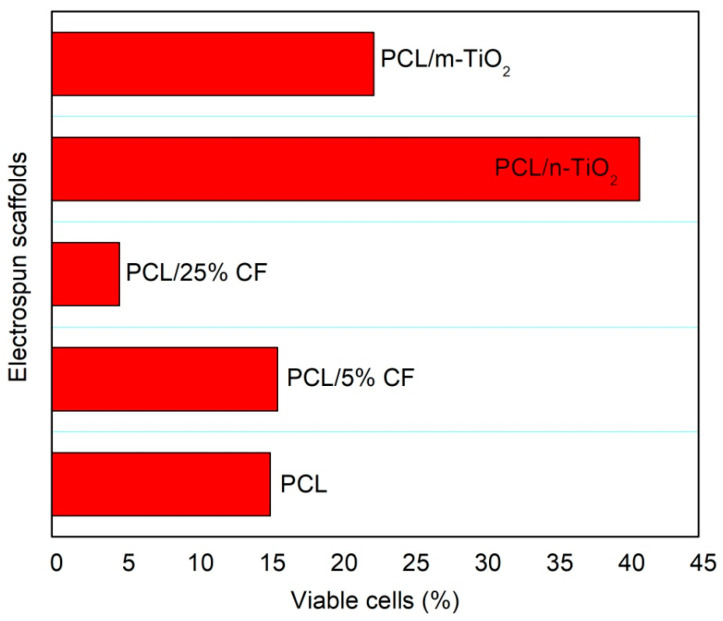
Percentage of viable cells measured by the 3-(4,5-dimethylthiazol-2-yl)-2,5-diphenyltetrazolium bromide (MTT) assay.

All scaffolds showed that the scaffolds’ surfaces were partially or fully covered by the adhered LSCs. In terms of LSCs shapes the cells were joined and have shown elongated and flat conformation. Figure 15 presents the percentage of viable cells, measured by the MTT assay and survived onto the electrospun scaffolds surfaces. The highest percentage of viable cells (~40%) was calculated for the PCL/n-TiO_2_ scaffold, following PCL/m-TiO_2_ scaffold with ~20%, while all other scaffolds have shown a percentage of viable cells below 15%. The lowest percentage of survived cells was calculated for the electrospun scaffold, with the highest cefuroxime concentration (below 5%).

### 3.8. Immunocytochemistry (ICC) of the LSCs Cultured on the Electrospun Scaffolds

**Figure 16:**
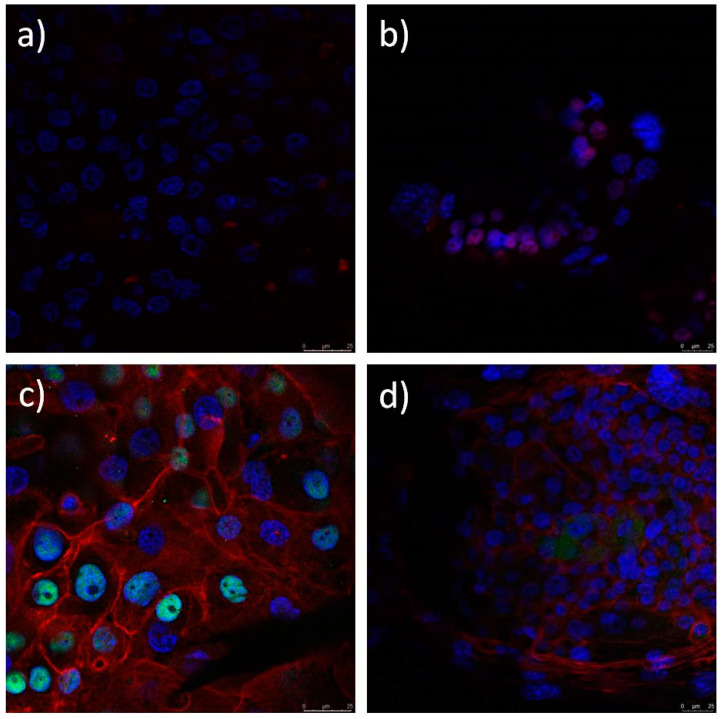
Immunofluorescence analysis of limbal stem cells cultured on PCL scaffold. Cells positive on cornea marker CK3 have cytoplasm coloured red (**a**,**b**) and cell positive on stem cell marker p63 show nuclei stained with turquoise. Cytoskeleton is stained red with phalloidin- tetramethylrhodamine B isothiocyanate (TRITC) (**c**,**d**). All nuclei are counterstained with blue stain 4′,6-diamidino-2-phenylindole (DAPI).

**Figure 17:**
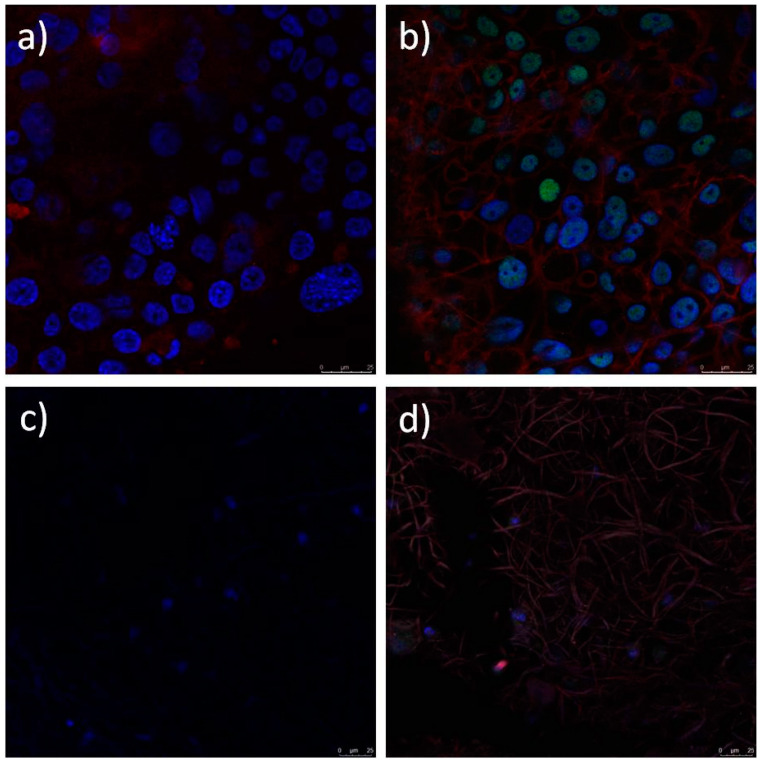
Immunofluorescence analysis of limbal stem cells cultured on PCL/5 wt % CF (**a**,**b**) and PCL/25 wt % CF (**c**,**d**) scaffold. Cells stained against cornea marker CK3 have cytoplasm coloured with red (**a**,**c**). Cell stained against stem cell marker p63 show nuclei stained with turquoise. Cytoskeleton is stained red with phalloidin-tetramethylrhodamine B isothiocyanate (TRITC) (**b**,**d**). All nuclei are counterstained with blue stain 4′,6-diamidino-2-phenylindole (DAPI).

**Figure 18:**
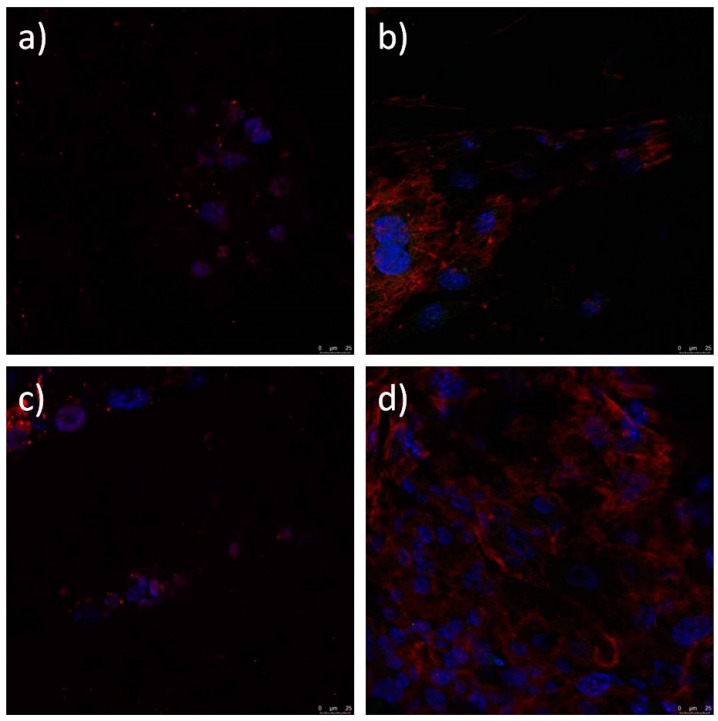
Immunofluorescence analysis of limbal stem cells cultured on PCL/n-TiO_2_ (**a**,**b**) and PCL/m-TiO_2_ (**c**,**d**) scaffold. Cells stained against cornea marker CK3 have cytoplasm coloured with red (**a**,**c**). Cell stained against stem cell marker p63 show nuclei stained with turquoise. Cytoskeleton is stained red with phalloidin-tetramethylrhodamine B isothiocyanate (TRITC) (**b**,**d**). All nuclei are counterstained with blue stain 4′,6-diamidino-2-phenylindole (DAPI).

Limbal stem cells cultured on the electrospun PCL, PCL/5 wt % CF, PCL/25 wt % CF, PCL/n-TiO_2_ and PCL/m-TiO_2_ scaffolds, were evaluated with immunofluorescence staining for the presence of limbal stem cell p63 marker, a nuclear transcription factor and cytokeratin 3 (CK3), a corneal epithelial differentiation marker. Figure 16, Figure 17 and Figure 18 present the confocal images of the LSCs onto the electrospun PCL, PCL/CF and PCL/TiO_2_ scaffolds, respectively, after immunofluorescence staining. The LSCs were identified as positive on cornea marker CK3, when their cytoplasm was coloured red. Their nuclei was coloured turquoise when the cells were positive on stem cell marker p63 and the cytoskeleton was red. All cells nuclei were counterstained with blue.

## 4. Discussion

Generally, electrospun scaffolds simulate the extracellular matrix or the native surrounding of the cells in human tissues, where the scaffolds microstructure and fibres morphology should be similar to the ones of the native tissues, in order to facilitate cells to attach and stimulate further growth. This study focuses on scaffolds to be potentially used in ocular tissue repair. By composition, the cornea’s major layer (out of five) overtaking 90% of the cornea is the stroma, consisting of collagen type I fibrils. These fibrils are uniform along the length, densely packed in parallel to form multiple lamellae. The orientation of the fibrils varies in the adjacent lamellae, providing elegant ultrastructural organization and resulting in high isotropic strength [53]. Another important property of the cornea, coming from the corneal epithelium layer, is the secretion of anti-inflammatory and anti-microbial factors for tear fluid layer stabilization, thus outer surface protection [54]. Usually, the outermost epithelial layer is the one that is most easily injured and having healthy limbal stem cells is of paramount importance for its renewal [55]. In this study, the fabricated scaffolds tend to mimic to some extent the structure of the target, as the patterned 3D printed collector used, provides specific ribbed topography, Figure 2, thus mimicking the parallel fibrils. The collector assisted in the construction of the 3D scaffold structure. On the other hand, the active antimicrobial components, the CF and the TiO_2_, tend to simulate the corneal secretion effect. The major requirement in scaffold development is its physical structure and mechanical integrity, as well as specific active components function, in this case the antimicrobial efficiency.

In terms of physical structure evaluation, this study have considered scaffolds fibres morphology, porosity and tensile properties. The fibres diameter was affected by the scaffolds composition, thus the addition of the antibiotic has resulted in significant reduction of the PCL 374 nm mean fibre diameter, by 36% in case of the 5 wt % CF, while the opposite effect was observed in case of the 25 wt % CF (thicker mean fibre diameter, increase by almost threefold). In electrospinning, the fibre’s diameter can be affected by a change in solution concentration (or viscosity), as well as conductivity, which in this case depends on, for example, drug size and chemical composition, thus interaction with the polymer [56]. Cefuroxime is a small drug molecule and as reported [56], small molecule might interrupt chain entanglement resulting in fibre diameter decrease. On the other hand, the effect observed for the PCL/25 wt % CF scaffold, can be explained namely by the polymer-drug interaction and the high CF concentration, which increases solution viscosity and results in higher fibre diameter. The addition of the TiO_2_ particles have both (nano and micro) increased fibre diameter up to 516 nm, which can be explained by the ultrasonic treatment and incorporation of the particles. These results are in compliance with the scaffolds’ calculated porosity. Generally, higher fibre diameter results in higher scaffolds porosity, as finer fibres tend to accumulate more compactly [57]. The initial porosity of the PCL scaffolds was calculated to be 86.80 ± 1.05%, as the fibres’ diameters decreased with the addition of the 5 wt % CF, the total porosity decreased as well (78.58 ± 1.45%). All other scaffolds that were observed to have higher fibre diameters, were also calculated to have increased porosities for up to 92.87 ± 3.13%. Porosity is directly related to scaffolds permeability/fluid mobility, which are further affecting nutrient and waste transport within the structure [58]. From this point of view, the developed electrospun structures fully satisfy scaffolds primary requirements, as all scaffolds have shown total porosity in the ranges of almost or above 80%, Figure 5.

Another important requirement in scaffolds development is their mechanical integrity, which needs to be satisfactory, in order for the scaffold to withstand the forces that are acting on the structure during tissue formation and growth [59]. In regard to scaffolds mechanical performance, the results suggest that the addition of the CF and TiO_2_ into the PCL, directly influences scaffolds elasticity [60] and that almost all scaffolds have very close values of their tensile strengths, Table 2, to the mean value of the tensile strength in human cornea, as reported of 3.8 MPa [61]. The forth-mentioned, two physical parameters, can be further related to the scaffolds tensile stress performance, Figure 7. The change of the fibre’s diameter correlates to the change in the scaffolds tensile strength, Table 2. Thicker fibres resulted in fibrous scaffolds higher tensile strength, that is, the highest tensile strength was in case of the PCL/25 wt % CF scaffold, thus the ~3.5 N/mm^2^ has corresponded to the highest fibre mean diameter of 1 micron. In case of the scaffolds total porosity, a general assumption would be that with the increase of the porosity, there will be a decrease in the tensile strength [62]. In this study both effects were observed, as in case of the TiO_2_ based scaffolds, the higher porosity was correlated to slightly lower tensile strengths bellow 3 N/mm^2^. On the other hand, lowest porosity which was in case of the PCL/5 wt % CF scaffold, also resulted in lowest tensile strength of ~0.7 N/mm^2^. These results can also be further related to scaffold’s measured thickness, which in case of the PCL/5 wt % CF scaffold was measured to be the lowest, thus of ~100 microns.

The Young’s modulus is the measure of the scaffold’s stiffness. It seems, that the higher the CF content was in the PCL, the higher the scaffolds stiffness was observed, which is strongly evident in case of the electrospun PCL/25 wt % CF scaffold (23.26 ± 6.08 MPa at 5% strain), compared to the pure PCL (0.62 ± 0.08 MPa at 5% strain). On the other hand, the low 5 wt % CF content has not compromised the polymer elasticity, thus at the strain level of 10%, the mean Young’s modulus for this scaffold was calculated to be 1.14 ± 0.26 MPa. These results are also related to the maximum and minimum mean values of the scaffold’s fibre diameters, which seems to comply with the high increase of the Young’s modulus [63]. The observations may also be related to the degree of crystallinity as previously reported [26]. It was suggested, that the higher content of CF increased the degree of crystallinity of the scaffolds. Thus, in high performance plastics the crystalline phase is the one that is responsible for the increase in stiffness and tensile strength [64]. Extreme increase in the Young’s modulus was also reported in the fabrication of elastin/poly-lactic-co-glycolic acid (PLGA) scaffolds in case of elastin surface modification (covalent bonding), resulting in mean values of 20.72 ± 5.978 MPa, compared to the pure PLGA of ~0.59 ± 0.356 MPa [65].

The functional role of the scaffolds in this study is their antimicrobial activity, against the three pathogens (*P. aeruginosa*, *S. aureus and C. albicans*), that are frequently causing eye problems. The antimicrobial activity was confirmed, Table 3, Figure 11 and Figure 12, although two types of tests were conducted specifically for the PCL/m-TiO_2_ scaffold. The variation in the response of the micro TiO_2_ based scaffolds, is generally related to the particles possible inhomogeneous deposition on the surface of the scaffolds, due to its agglomerations, Figure 3e, formation during the sonication process. To lessen the consequence of serious postoperative infections, systemic and topical antibiotics can be applied postoperatively but that has downsides [66,67]. Systemic administration effects the whole organism but has limited capacity to reach target site. On the other hand, antibiotic eye drops act only for a limited time and cannot deliver drug in a preferable continuous fashion. It is reasonable to assume, that PCL with the incorporated antimicrobial components, cefuroxime and TiO_2_, should have an advantages as LSCs carrier in clinical setting. It can exhibit a potent antimicrobial activity against bacteria that often cause serious eye infections. What is more important, PCL scaffolds are able to continuously release these antibiotic compounds in a time frame for a carrier to be partially or completely dissolved. Scaffolds release behaviour, Figure 13, is directly related to the active particles’ encapsulation within the polymer matrix. In case of the TiO_2_ the released amounts were very low to be detected, due to the weak coating or inhomogeneous distribution of the particles. The burst release phase of the CF is suggested to be due to the possible presence of the drug on the surface of the fibres [68], which is probably higher in case of the higher CF concentrations, thus the 25 wt %. The drug surface presence is usually a result of a non-perfectly encapsulated component inside the fibres. These results suggest very low overall release contents of the CF which might be due to the possible drug polymer interactions [68]. Another reason might be the PCL hydrophobicity in case of the PCL/5 wt %, which may slower the diffusion of the CF due to the longer time needed for the PBS to wet the scaffold surface. This type of drug’s slow release profile may be adequate in case of continuous long term patient therapy needed.

Electrospun scaffolds are very much explored as artificial supports of living cells and generally corneal regeneration is considered through animal limbal model cells [69]. In this study, human limbal stem cells were successfully cultivated on all electrospun PCL, PCL/CF and PCL/TiO_2_ scaffolds tested, as seen on the SEM and confocal images, Figure 14 and Figure 16, Figure 17 and Figure 18, respectively. Lesser coverage of the scaffolds surface, Figure 14, confirms the sensitivity of these cells and their necessary period to accommodate in a new environment. Extremely low and extremely high viable cells percentage, Figure 15, were noticed for the PCL/5 wt % CF and PCL/n-TiO_2_ scaffolds, respectively, which was to some extent in compliance with the immunofluorescence analysis, Figure 16, Figure 17 and Figure 18, although the latter is more valid as it gives an insight into the type of survived cells.

Identification of cultured human LSCs and their differentiation is important, as reported, long-term stable LSCs transplantation (rate of ~80%) is associated with more than 3% of cells identified with the p63 marker [70]. In this study the immunofluorescence analysis has confirmed that all scaffold types support the stem cell-like (p63-positive) and cell corneal epithelial differentiation (CK3-postive), with an advantage of the PCL and the PCL/5 wt % CF scaffolds, as was observed by the cells confocal identification. This could be explained with possible cytotoxicity of the scaffolds’ active compounds or more precisely with their set concentration. It is known that the antimicrobial effect of TiO_2_ particles is higher when the same are activated by an UV light during bacteria diffusion [37]. This might potentially increase safety issue, as the photocatalytic effect on TiO_2_ particles can compromise not only limbal cells but also host cells viability following transplantation. On the other hand, extremely high cefuroxime content might have a negative effect on cells normal proliferation and differentiation, thus an optimum content would be beneficial, in order to keep cells alive and safe from usual contaminates. The next step in this research would be to overcome the limitation of the study, which concerns the exact number of LSCs and their ability to differentiate into corneal epithelial cells, thus, to quantify the expression of the p63 marker in order to evaluate cell stemness.

## 5. Conclusions

The study focuses on the development of electrospun PCL/CF and PCL/TiO_2_ scaffolds with an embedded antimicrobials against common pathogens causing eye infections, to support the adhesion, proliferation and growth of cultivated limbal stem cells for corneal tissue regeneration. The scaffolds were evaluated in regard to physical structure, wettability, mechanical performance, antimicrobial component release and efficacy in addition to the limbal cell growth/differentiation support. The addition of the 25 wt % CF and the TiO_2_ particles resulted in the increase of the fibre diameter, within the range of 450 nm to 1 μm. The increase in the scaffolds total porosity, was generally related to the increase in the fibre diameter, with the maximum calculated to be ~93% for the PCL/n-TiO_2_ scaffold. The other scaffolds also provided high total porosity important for nutrient and cells waste transport. All electrospun scaffolds, excluding the PCL/5 wt % CF scaffold, have shown a tensile strength close to the mean value of the tensile strength in human cornea. Extremely high Young’s modulus was observed for the PCL/25 wt % CF scaffold, which can be related to thicker fibres and a higher degree of crystallinity. The electrospun PCL/5 wt % CF and PCL/m-TiO_2_ scaffolds exhibited statistically significant antimicrobial activity when compared to pure PCL scaffold against *C. albicans* and *P. aeruginosa*. The CF release profile showed initial burst effect, followed by the slow sustained release in optimal concentrations, adequate for a continuous long term post-transplant therapy. Immunocytochemistry revealed both limbal stem cells and differentiated corneal cells on all electrospun scaffolds, positive on p63 marker, thus coloured with red cytoskeleton and turquoise nuclei or positive on cytokeratin CK3 marker, thus coloured with red cytoplasm and blue nuclei, respectively. The advantage was mostly seen for the electrospun PCL and PCL/5 wt % CF scaffolds, in terms of cell type identification (differentiated corneal and stem cell-like cells). The reason for these results is explained with the possible cytotoxicity of scaffolds’ active compounds, that have to have a limit content as not to compromise specific cell type viability.

## Figures and Tables

**Table 1:** Water contact angle measured on the surface of the electrospun scaffolds.

Electrospun Scaffolds	Measured Water Contact Angles(◦)	
	1 s	3–5 s
PCL	117.8 ± 4.4	117.8 ± 4.4
PCL/n-TiO_2_	123.8 ± 2.6	122.6 ± 3.9
PCL/m-TiO_2_	124.4 ± 7.7	122.9 ± 5.0
PCL/5 wt % CF	112.0 ± 5.4	86.7 ± 16.1
PCL/25 wt % CF	-	-

**Table 2:** Maximum force, elongation at break and tensile strength of the electrospun scaffolds.

Electrospun Scaffolds	F_max_ (N)	ɛ (%)	σ (N/mm^2^)
PCL	5.66 ± 1.40	169.50 ± 26.15	3.41 ± 0.84
PCL/5 wt % CF	0.72 ± 0.09	38.50 ± 3.77	0.68 ± 0.09
PCL/25 wt % CF	5.03 ± 0.51	364.00 ± 32.32	3.53 ± 0.34
PCL/n-TiO_2_	4.22 ± 0.33	110.00 ± 9.04	2.58 ± 0.20
PCL/m-TiO_2_	4.08 ± 0.25	150.50 ± 12.12	2.52 ± 0.15

**Table 3:** Inhibition zone in mm.

		Inhibition Zone (mm)		
Electrospun Scaffold	Label	*P. aeruginosa*	*S. aureus*	*C. albicans*
PCL	0	n.p.	n.p.	n.p.
PCL/5 wt % CF	4	27.0 ± 1.41	31.0 ± 1.41	n.p.
PCL/25 wt % CF	3	40.5 ± 0.71	44.0 ± 1.41	n.p.
PCL/n-TiO_2_	2	18.5 ± 2.12	n.p.	n.p.
PCL/m-TiO_2_	1	n.p.	n.p.	n.p.

n.p.—not proven.
